# Willingness of secondary school adolescents to get vaccinated against Malaria: a cross-sectional study in Enugu, South-East Nigeria

**DOI:** 10.4314/ahs.v25i2.9

**Published:** 2025-06

**Authors:** Obinna C Nduagubam, Edmund N Ossai, Awoere T Chinawa, Vivian O Onukwuli, Ndubuisi A Uwaezuoke, Chinyere N Okafor, Josephat M Chinawa

**Affiliations:** 1 Department of Paediatrics, College of Medicine, Enugu State University of Technology, Enugu State; 2 Department of Community Medicine, Ebonyi State University, Abakaliki, Ebonyi state; 3 Department of Community Medicine, College of Medicine, Enugu State University of Technology, Enugu; 4 Department of Paediatrics College of Medicine, University of Nigeria, Ituku-Ozalla, Enugu State; 5 Department of Community Medicine, College of Medicine, University of Nigeria, Ituku-Ozalla, Enugu State

**Keywords:** Willingness, malaria vaccine, secondary school adolescents, Knowledge

## Abstract

**Background:**

Malaria is a serious public health challenge both in children and adolescents.

**Objectives:**

This study was aimed to document the willingness of the adolescents to accept vaccine and its associated factors.

**Methodology:**

This was an observational and cross-sectional study on four hundred and ninety-six secondary school adolescents in six secondary schools in Enugu from June 2023 to September 2023. Data entry and analysis were done using IBM Statistical Package for Social Sciences (SPSS) statistical software version 25.

**Results:**

A reasonable proportion of the respondents, 68.1% were willing to receive malaria vaccine. A higher proportion of the respondents, 59.3% knew that malaria vaccination could protect against seasonal influenza. Less than one third of the respondents, 32.1% had good knowledge of malaria vaccination. The respondents who were less than 15 years were twice more likely to receive malaria vaccination when compared with those who were 15 years and above, (AOR=2.2, 95%CI: 1.1-4.7). The respondents who were males were about twice less likely to receive malaria vaccination when compared with those who were females, (AOR=0.6, 95%CI: 0.4-0.9). The respondents who had good knowledge of malaria vaccination were twice more likely to receive malaria vaccination when compared with those who had poor knowledge, (AOR=2,2, 95%CI: 1.4-3.4)

**Conclusion:**

A reasonable proportion of secondary school adolescents were willing to receive the malaria vaccine. Willingness to receive malaria vaccine is influenced by gender, knowledge of malaria vaccine and age of the adolescent.

## Introduction

Malaria constitutes a serious public health challenge with about 4 billion people at risk of infection^1^. It is a major cause of under-5 morbidity and mortality^1^,^2^. Recently, due to the concentration of attention and efforts at fighting the COVID-19 pandemic, the overall prevalence and mortality rate from malaria infestation has risen to 241 million and 627,000 respectively in the year 2020^2^,^3^. This results in an increased prevalence of 14 million cases and 69,000 deaths when compared to the previous years^3^. Though Malaria severity and complications occur mainly in the under-fives, school age children and adolescents can become vulnerable especially if the population has high exposure to infection, low level of acquired immunity and malnutrition^4^. In areas of high malaria transmission, younger children are the bane of infection^4^,^5^. However, areas with good control of malaria have a remarkedly reduced level of transmission^6^,^7^. In such region the age prevalence has shifted from the under-fives to the adolescents. For instance, the peak incidence of malaria infection has increased by 1.7 years in about 4 years^8^,^9^. Similar changes documented in the Kenya study^7^.

The burden of malaria among older children and adolescents is not well known, as this age group is not routinely included in malaria prevention surveys. Data on the burden and prevalence of malaria is derived mainly from school-based studies as well as from World Health Organization (WHO) estimates^8^. Gething^9^ et al documented that the world has recorded more than 500 million school-age children/adolescents with malaria infection of which about half of these values are seen majorly in Sub-Saharan Africa. Several methods have been employed in the prevention of malaria^10^. These include the use of insecticide-treated nets, reduction of insect breeding sites, indoor residual spraying, intermittent preventive treatment, and intermittent screening and treatment^10^-^14^. However, recently, the world's first vaccine against malaria was developed and was approved for developing countries where malaria accounts for 1 in 20 human deaths^15^. The World Health Organization (WHO) has recommended that children and adolescents who live in regions with a high transmission rate should receive the RTS, S/AS01e vaccine against the Plasmodium falciparum^15^. The development of a malaria vaccine with proven efficacy has become a major issue, despite several innovative mechanisms for malaria vaccinology. For instance, vaccine (RTS, S/AS01) has shown partial efficacy in a large-scale Phase 3 clinical trial, besides, the duration of protection provided by RTS, S/AS01 is short^16^-^20^. Very few works and limited data are available on the safety and immunogenicity of RTS, S/AS01 in school-age children and adolescents^17^. However, the willingness to accept the Malaria vaccine among adolescents has remained obscure, due to the fact that most studies on malaria vaccinology were mainly among the under-fives^18^-^20^.

Though, the number of severities of malaria parasitemia per annum declines dramatically, especially after the age of peak prevalence and in adolescents when severe disease rarely occurs^21^. Yet clinical scenarios have been reported with a total prevalence of parasitaemia approaching 100% in adolescents^22^,^23^. Despite the fact that immunity provides defense against morbidity and mortality resulting from complications of malaria, adolescents still suffer some mild to moderate forms of malaria infection especially those who live in areas of hyper- to holo-endemicity^21^,^23^. For instance a study in a holoendemic northern city of Ghana showed that 2% of adults in a cohort of 192 population had an initial parasitaemia of more than >20,000 parasites/µl, with 97% of adolescents showing parasitaemia during the 16 weeks of follow-up^21^-^23^. The risk of morbidity and mortality among adolescents should not be downplayed especially those who are not exposed to the infection. Furthermore, there is no clear-cut estimate of the risk of mortality and morbidity in any age group with P. falciparum without any immunity or chemotherapy^19^,^20^. It is now documented that about 30% risk of death is reported among children and young adults without acquired malaria immunity or adequate chemotherapeutic management of malaria^23^.

The importance of adolescent uptake of malaria vaccine is crucial since there is no mechanistic or conclusive proof that demonstrates age-related differences in host susceptibility and responses to malaria infectious diseases due to its heterogeneity. Indeed, literature has shown that no study provided a succinct molecular or cellular mechanism that could explain or unravel these age-related phenomena^22^,^23^. Vaccine hesitancy has been documented in literature, and the willingness to accept malaria vaccine among children in low- and middle-income countries remains sub-optimal^19^. This study aimed to document the willingness of adolescents to accept malaria vaccine as well as its associated factors. This would help to establish the perception of the adolescents on malaria vaccine and their willingness to accept it.

## Methods

### Study Area

The study was carried out in ten secondary schools drawn from six rural and four urban schools located in Enugu.

### Study Design

This is an observational cross-sectional study conducted among ten secondary school adolescents from six rural and four urban secondary schools in Enugu, South East Nigeria.

### Study population

Four hundred and ninety-six students who were adolescents and who attended urban and suburban secondary schools were consecutively enrolled in the study.

### Inclusion Criteria

Adolescents who attended either urban or rural secondary school in Enugu and who gave consent/assent were included in the study.

### Exclusion Criteria

Adolescents without any form of consent were excluded from this study

### Sampling technique

Students included in this study were assessed using a two-stage sampling technique. There are four hundred and sixty-eight (468) secondary schools in Enugu metropolis. There are three municipal zones in the city. This included Enugu South, Enugu East and Enugu North municipal centres. The list of secondary schools in the municipal centres was drawn up and graded by the number of students in the secondary schools. The first ten schools based on the ranking in the three areas were selected. The first stage of selection was made by selecting one rural and one urban secondary school in each of the three municipal areas using a simple random sampling technique. Thereafter, list of secondary school was made, this is the second stage. On each day of questionnaire and data collection, the number of adolescents in the four classes from the selected ten schools served as the sampling frame. When we divide this number by the sample size of 200, we obtained the sampling interval.

### Study tool

A pre-tested, validated, self-administered questionnaire was used to obtain data from the adolescents in junior and senior secondary classes in the selected schools

### Sample Size Determination

With 10% attrition, a minimum sample size of 496 was obtained using the method of sample size estimation by Israel et al^25^. Here, a 95% confidence level and 5% precision for a population >100,000 for the study was used^25^.

### Definition of terms

**Adolescence:** This is a period of transition between childhood and adulthood.

**Willingness to be vaccinated:** This is defined as the preparedness or readiness to receive a vaccine after considering the efficacy, benefits, adverse effects, and barriers that may be associated with receiving such vaccine^27^.

### Data analysis

Data entry and analysis were done using IBM Statistical Product and Service Solutions (SPSS) statistical software version 25. Categorical variables were described using frequencies and proportions while continuous variables were presented using mean and standard deviation. Chi square test and multivariate analysis using binary logistic regression were used in the analysis and the level of statistical significance was determined by a p value of <0.05. The outcome variable was willingness to receive malaria vaccine among the respondents. This was assessed using a single variable, ‘Do you intend to receive malaria vaccine.’ In determining the factors associated with willingness to receive malaria vaccine, the socio-demographic and socio-economic characteristics of the respondents were cross-tabulated with the outcome variable. In determining the predictors of the outcome variable, variables that had a p value of <0.2 on bivariate analysis were entered into the logistic regression model. The results of the logistic regression analysis were presented using adjusted odds ratio and 95% confidence interval and the level of statistical significance was determined by a p value of <0.05. Knowledge of malaria vaccine was determined using nine variables. For each variable, the respondents were awarded a score of one for a correct response and a sore of zero for an incorrect response. Respondents that scored ≥50% of the total variables were regarded as having good knowledge of malaria vaccination while those that scored <50% were regarded as having Poor knowledge of malaria vaccination. The socio-economic class of the family of the respondents was determined using the Oyedeji classification

## Results

Table 1 shows the socio-demographic characteristics of the respondents. The mean age of the respondents was 15.1±1.7 years and a higher proportion, 70.8% were 15 years and above. Majority of the students, 61.7% were females. The highest proportion of the students, 28.0% were in senior secondary one class while the least proportion, 0.6% were in junior secondary three class.

**Table 1:** Socio-demographic characteristics of the respondents

Variable	Frequency (n=496)	Percent (%)
**Age of respondents**		
Mean±SD	15.1±1.7	
		
**Age of respondents in groups**		
<15 years	145	29.2
≥ 15 years	351	70.8
		
**Gender**		
Male	190	38.3
Female	306	61.7
		
**Educational level**		
Junior Secondary 1 class	37	7.5
Junior Secondary 2 class	81	16.3
Junior Secondary 3 class	3	0.6
Senior Secondary 1 class	139	28.0
Senior Secondary 2 class	137	27.6
Senior Secondary 3 class	99	20.0
		
**Family Socio-economic Class**		
High socio-economic class	150	30.2
Middle socio-economic class	171	34.5
Low socio-economic class	175	35.3

Table 2 shows the willingness to receive malaria vaccine among the respondents. A higher proportion of the respondents, 68.1% were willing to receive malaria vaccine.

**Table 2:** Willingness to receive malaria vaccine among the respondents

Variable	Frequency (n=496)	Percent (%)
**Willingness to receive malaria vaccine**		
Yes	338	68.1
No	158	31.9

Table 3 shows the knowledge of malaria vaccination among the respondents. Less than half of the respondents, 44.9% were aware that adolescents are not among the high priority group for malaria vaccine in Nigeria. A higher proportion of the respondents, 59.3% knew that malaria vaccination could protect against seasonal influenza. A minor proportion of the respondents, 47.8% regarded malaria vaccine as being beneficial. Less than one third of the respondents, 32.1% had good knowledge of malaria vaccination.

**Table 3:** Knowledge of malaria vaccination among the respondents

Variable	Frequency (n=496)	Percent (%)
**Included in high priority group for vaccination**		
Yes	274	55.2
No (correct)	222	44.8
		
**There are much more risks of side effects from malaria vaccination**		
Yes	361	72.8
No (correct)	135	27.2
		
**Malaria vaccination could protect against seasonal influenza**		
Yes (correct)	294	59.3
No	202	40.7
		
**Most reactions to malaria vaccination are mild**		
Yes (correct)	188	37.9
No	308	62.1
		
**Malaria vaccination could cause influenza**		
Yes (correct)	73	14.7
No	423	85.3
		
**Most reactions to malaria vaccination do not last long**		
Yes (correct)	235	47.4
No	261	52.6
		
**Vaccinations could cause side effects**		
Yes (correct)	116	23.4
No	380	76.6
		
**Believe that malaria vaccination is safe**		
Yes (correct)	305	61.5
No	191	38.5
		
**Malaria vaccination is beneficial**		
Yes (correct)	237	47.8
No	259	52.2
		
**Knowledge of malaria vaccination**		
Good	159	32.1
Poor	337	67.9

Table 4 shows the factors associated with willingness to receive malaria vaccine among the respondents. Students younger than 15 years of age 113 (77.9) are more willing to receive malaria vaccine than those above 15 years of age 225 (64.1). P= 0.03. Male adolescents are more willing to receive vaccine 116 (61.1%) than their female counterparts 222 (72.5%). P=0.008. One hundred and twenty-five (786%) had good knowledge of malaria vaccination.

**Table 4:** Factors associated with willingness to receive malaria vaccine among the respondents

Variable	Willingness to receive malaria vaccine (n=496)			
	Yes N (%)	No N (%)		
**Age of respondents in groups**				
<15 years	113 (77.9)	32 (22.1)	9.039	0.003
≥15 years	225 (64.1)	126 (35.9)		
				
**Gender**				
Male	116 (61.1)	74 (38.9	7.137	0.008
Female	222 (72.5)	84 (27.5)		
				
**Educational level**				
Junior Secondary Class	92 (76.0)	29 (24.0)	4.587	0.032
Junior Secondary 2 class	246 (65.6)	129 (34.4)		
				
**Family Socio-economic Class**				
High socio-economic class	99 (66.0)	51 (34.0)	0.457	0.796
Middle socio-economic class	118 (69.0)	53 (31.0)		
Low socio-economic class	121 (69.1)	54 (30.9)		
				
**Knowledge of malaria vaccination**				
Good	125 (78.6)	34 (21.4)	11.820	0.001
Poor	213 (63.2)	124 (36.8)		

Table 5 shows the factors associated with willingness to receive malaria vaccination among the respondents. The respondents who were less than 15 years were twice more likely to receive malaria vaccination when compared with those who were 15 years and above, (AOR=2.2, 95%CI: 1.1-4.7). The respondents who were males were about twice less likely to receive malaria vaccination when compared with those who were females, (AOR=0.6, 95%CI: 0.4-0.9). The respondents who had good knowledge of malaria vaccination were twice more likely to receive malaria vaccination when compared with those who had poor knowledge, (AOR=2,2, 95%CI: 1.4-3.4)

**Table 5:** Predictors of willingness to receive malaria vaccine

Variable	Adjusted odds ratio	P value	95% Confidence Interval	
			Lower	Upper
**Age of respondents in groups**				
<15 years	2.221	0.038	1.0437	4.72
≥ 15 years	1			
				
**Gender**				
Male	0.620	0.017	0.4199	0.91
Female	1			
				
**Educational level**				
Junior Secondary Class	0.862	0.714	0.390	1.905
Junior Secondary 2 class	1			
				
**Knowledge of malaria vaccination**				
Good	2.167	0.001	1.3885	3.38
Poor	1			

## Discussion

Malaria vaccine uptake is a common topical issue in our setting. Most of the work on malaria vaccinology is mainly among children. Among secondary school adolescents, malaria causes school absenteeism, recurrent failures in termly exams, poor performance in school, and high level of school dropouts with a very high mortality. Several reports had shown that over 60% of Intelligent quotients had been impaired by malaria^28^-^30^. The burden of malaria parasitemia among secondary school adolescents cannot be overemphasize and this had been shrouded by the eclipse of Pediatrics HIV and AIDS^30^.

This work has shown that only 32.1% of secondary school adolescents were aware of the availability of malaria vaccine. This poor knowledge could be due to few (55.2%) adolescents who accepted the fact that they should be included in high priority group for vaccination and 38.5% of them even believed that malaria vaccination is unsafe.

The low prevalence obtained from this study may also be explained by the low knowledge of malaria infection among them. For instance, Sumari et al^31^ in India noted that nearly half (51.1%) of the adolescent had knowledge of malaria transmission^31^. Initially, development of a very potent malaria vaccine has proven a major challenge, however currently, vaccination is a very crucial element used for the prevention of malaria in secondary school children^32^

Careful search in literature had shown no work on malaria vaccine uptake among adolescents and this could be explained by the neglect of adolescents' health and well-being.

During the COVID 19 pandemic, the vaccine uptake in adolescents were very low. For instance, Chinawa et al^33^ noted an uptake of 13.2% while Olu-Abiodun et al^34^ documented an uptake within the range of 20-58.2%. The reason for this low uptake was attributed to the fact that low priority for COVID 19 vaccination was given to adolescents. However, the burden of malaria infection is quite higher compared to that of COVID 19. Much attention should be given towards the prevention of malaria infection.

Weiss et al^35^ has noted that good progress was achieved in the first two decades in reducing the burden of malaria in sub-Saharan Africa. However, this great milestone is jeopardised by the attention given to COVID-19 pandemic, which has affected the availability of malaria interventions using vaccine. They noted that the disruption in malaria control by COVID-19 may double malaria mortality in 2020^35^.

This study showed that 68.1% of secondary school adolescents are willing to receive the malaria vaccine. The suboptimal willingness to accept malaria vaccine in this study is due to the fact that 72.8% of adolescents noted that there are much more risks of side effects from malaria vaccination and 52.2% suggested that Malaria vaccination is not beneficial while 62.1% noted that most reactions to malaria vaccination are severe. Though the new Malaria vaccine may prevent complications from Malaria infections but it is not known if Malaria vaccination will curb the spread of the Malaria among the adolescents. Besides, it is also not known if adolescents who were vaccinated against malaria may be indolent spreaders of the parasite, since they may have the propensity to keep it in circulation in the communities, putting those without malaria vaccination at a greater risk as seen in the COVID 19 pandemic^36^,^37^

Besides, the malaria vaccine was initially procured for about 360 000 children each year across three countries with vaccination schedule based on WHO recommendations^38^. However the age limit excluded adolescents, as vaccination are limited to age of five years in Malawi and the age of six months in Ghana and Kenya. A fourth dose schedule is recommended at the second birthday^38^.

The major reason adolescents were unwilling to to receive the malaria vaccine as seen in this study were not being sure of the content of the vaccine, not being sure of the source of the vaccine, and fear of complications. Other reasons were infertility. These adolescents show uncertainties on the safety of malaria vaccine^38^. Mosquirix, the first licensed malaria vaccine, was given approval by the European Medicines Agency only for pilot use. This rose from the reports of various side effects as it was noted that the rate of meningitis among children who took Mosquirix was about 10 times of those who did not receive the vaccine^38^. Furthermore, increased cerebral malaria episodes and increased number of deaths had occurred^38^.

This current study had shown that secondary school adolescents who had good knowledge of Malaria vaccination were twice more likely to receive the Malaria vaccination when compared with those who had poor knowledge. This poor knowledge is also corroborated by Nnaji et al^39^ who noted that in Nigeria, malaria prevention measures, only consist of the use of insecticide-treated bed nets and ACT^40^, but this is the same with countries like Ghana, and Kenya that have implemented malaria vaccine programmes. Nnaji et al^39^ also noted in their study that only (48.9%) of the subjects are aware of the malaria vaccine. While 67.8% noted malaria vaccination as one of the control measures in Nigeria.

The current study showed that adolescents who are younger than 15 years (77.9 %) were more willing to receive malaria vaccine than their older counterparts (64.1%). The younger adolescents also have about twice odds of willingness to accept the malaria vaccine than their older counterparts. The hesitancy among the older adolescents is also buttressed by the study of Ishimaruet al^41^ who noted that the older the respondents had higher the odds that they will less likely know and accept vaccination policy. This finding may be explained by the fact that education and awareness on the malaria vaccine policy are limited to mothers and the under five children and younger adolescents. Awareness of malaria vaccine uptake and the benefits should address health problems in the adolescent population and adolescent's uptake of malaria vaccine leads to reduced confidence in the statement that a new policy on malaria vaccine uptake among adolescence should be adopted in Nigeria.

This study had shown that female adolescents (72.5%) are willing to receive malaria vaccine compared with their male (61.1%) folk. Furthermore, male adolescents were about twice less likely to receive malaria vaccination when compared with those who were females. This finding negates the finding of Ishimaru et al who noted that though females tend to be more proactive in preventive behaviours, but they are less willing to accept vaccination than the males^42^.

A Chinese study had shown that the quest for men to get vaccinated could be explained by the findings in a recent study that showed that women's samples had more antibodies which may have an impact on their immune response compared to the men's samples^42^. Other studies have corroborated vaccine hesitancy to be higher in females than males^43^,^44^. This study however is not in consonance with that of Lazarus et al^44^ who noted no gender bias in their reports. Sample size and sociocultural differences could explain the varying findings. The female preponderance documented in this study could also be due to a higher number of female subjects recruited in the study.

The current study showed no significant difference in socioeconomic class and willingness of the adolescents to receive vaccine. This study is at variance with that of Bertoncello et al^45^ who noted a rising levels of perceived hardship and low socio-economic class as a reason for vaccine hesitancy.

Secondary school adolescents who had knowledge of being infected with Malaria were 2.167 more likely to receive Malaria vaccine than those who believe they cannot be infected. Misinformation and disinformation are strong tools for vaccine scepticism^46^. There is therefore clarion call for a thorough and effective dissemination of information on the role of malaria vaccine as a means of curbing malaria infection.

## Conclusion

A reasonable proportion of secondary school adolescents were willing to receive the malaria vaccine. Willingness to receive malaria vaccine was influenced by gender, knowledge of malaria vaccine and age of the adolescent.

## Recommendation

There is malaria vaccine hesitancy among secondary school adolescents. Effective dissemination of information on the role of malaria vaccine as a means of curbing malaria infection is crucial.

## Limitation

This work is limited by the fact that it was conducted in one state of the country. A nationwide study with cohorts of adolescents over a very long time is necessary

## Data Availability

Data are how ever available from the authors upon reasonable request and with permission of the corresponding Author
